# Autophagy modulation in gynaecologic oncology: insights into immune regulation and therapeutic potential

**DOI:** 10.3389/fimmu.2026.1737681

**Published:** 2026-06-15

**Authors:** Mingyu Huang, Defeng Zhao, Rui Xiong, Ruimin Yuan, Yumeng Lin, Yi Wang, Caijun Xiong, Lan Yuan, Zhongyu Han, Haoran Chen

**Affiliations:** 1 School of Acupuncture and Tuina, Chengdu University of Traditional Chinese Medicine, Chengdu, China; 2 Department of Thoracic Surgery, The First Hospital of China Medical University, Shenyang, China; 3 Science and Education Department, Chengdu Xinhua Hospital Affiliated to North Sichuan Medical College, Chengdu, China; 4 Department of Nanjing Tongren Eye Center, Nanjing Tongren Hospital, School of Medicine, Southeast University, Nanjing, China; 5 School of Medical and Life Sciences, Chengdu University of Traditional Chinese Medicine, Chengdu, China; 6 Zhongda Hospital, School of Medicine, Southeast University, Nanjing, China

**Keywords:** autophagy, cancer, drug resistance, gynaecological tumour, targeted therapy, tumour microenvironment

## Abstract

As a fundamental cellular process, autophagy maintains homeostasis and viability through the regulation of proteostasis, organelle quality control, and functional preservation. It represents the central pathway for transporting diverse cytoplasmic components to lysosomes for degradation and recycling. Accumulating evidence reveals the paradoxical role of autophagy in oncogenesis, where it can either suppress tumour development or facilitate cancer progression depending on context and stage. This conclusion is particularly evident during gynaecological tumour progression. Contemporary research priorities include deciphering the intricate involvement of autophagy in antitumour immunity and treatment resistance mechanisms. This comprehensive analysis systematically evaluates the dichotomous nature of autophagic processes across various malignancies and developmental phases, with a particular emphasis on how autophagy influences the dynamics of the gynaecological tumour microenvironment and modulates treatment responses. Key findings reveal that autophagy regulates immune checkpoint expression (e.g., PD-L1 and MHC-I); shapes antitumour immunity via T cells, macrophages, and other immune components; and modulates drug resistance through pathways involving AMP-activated protein kinase (AMPK), Heat shock factor 1 (HSF1), and Reactive oxygen species (ROS). These findings underscore the potential of autophagy as a therapeutic target and highlight strategies for combining autophagy modulators with conventional treatments to overcome resistance. This article provides a foundation for the development of precision medicine approaches tailored to autophagy-related pathways in gynaecologic malignancies.

## 1 Introduction

Global epidemiological data demonstrate that gynaecological neoplasms constitute a major public health burden. The incidence of cervical and uterine cancers is increasing, with advancing age and lifestyle determinants constituting prominent contributors to disease risk. Common clinical presentations of these malignancies include infertility and pelvic pain (1). Ovarian cancer (OC) is the deadliest gynaecologic malignancy, with 21,650 new cases in the U.S. annually and a 40–60% lifetime risk in BRCA1 mutation carriers (2). Clinically, these disorders exhibit distinct profiles, but from a prognostic perspective, they can damage fertility, mental health, and cardiovascular outcomes to some extent (2, 3). Critically, autophagy is associated with pathogenic process-specific modulation, revealing its therapeutic potential for these conditions.

**Figure 1:**
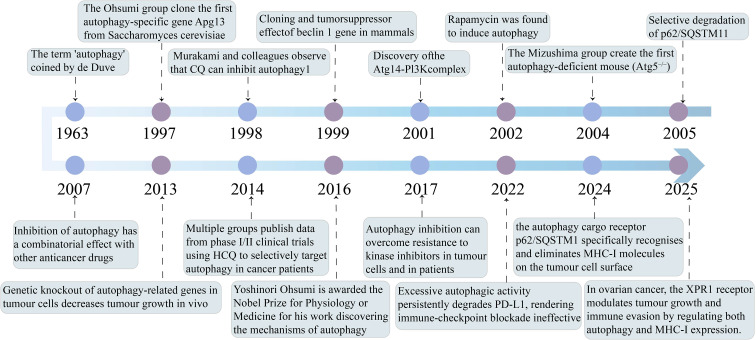
Timeline of pivotal autophagy discoveries and their applications in cancer. This timeline outlines pivotal findings in the field of autophagy and their relevance to tumour cells. Since Christian de Duve coined the term in 1963, landmark studies have elucidated its mechanisms, advancing from yeast and cellular models to mice. These studies ultimately revealed the critical roles of autophagy in maintaining homeostasis and facilitating pathological processes within tumour cells, culminating in successful clinical trials involving cancer patients. Due to space constraints, this compilation does not encompass all research achievements. (Atg, autophagy-related gene; CQ, chloroquine; HCQ, hydroxychloroquine; MHC-I, major histocompatibility complex I; PD-L1, programmed death ligand 1; PI3K, phosphoinositide 3-kinase; XPR1, xenotropic and polytropic retrovirus receptor 1; SQSTM1, sequestosome).

Since the initial characterization of autophagy in 1963, knowledge of its fundamental processes and therapeutic potential for enhancing patient care has evolved significantly (4) (**Figure 1**). As an evolutionarily conserved catabolic mechanism, autophagy facilitates lysosome-mediated macromolecule recycling to maintain intracellular equilibrium.

Autophagy is mainly divided into three distinct types: macroautophagy, microautophagy and chaperone-mediated autophagy (CMA), all three types play a role through unique mechanisms. Among them, the mechanism of macroautophagy is particularly important in the reproductive system (5). Macroautophagy involves the formation of autophagosomes with a double-layer membrane structure, which wrap proteins, organelles and other components in cell. Subsequently, the autophagosome fuses with the lysosome; the resulting hybrid organelle then breaks down the engulfed substrate through the action of hydrolases (6).

The tumour immune microenvironment (TME) constitutes a highly complex and dynamic ecosystem, which plays a decisive role in shaping the progression of cancer tumour cells evade immune surveillance through a variety of strategies, such as deleting or down-regulating tumour antigens to weaken immunogenicity and inhibiting immune signal transmission (7). In view of the profound impact of immunomodulation on tumour development and tumour growth, immunotherapy has become the cornerstone of modern anti-cancer treatment (8). Autophagy further regulates this microenvironment by reshaping the behaviour of tumour cells or immune cells and the secretion of cytokines, thus regulating the efficacy of immune response and anti-tumour immunotherapy (9).

This review analyses recent advances in autophagy research across gynaecological malignancies, integrating molecular mechanisms, signalling crosstalk and clinical impact. It delineates the pivotal influence of autophagy on neoplastic cell quality control and metabolic adaptation while providing a comprehensive overview of its emerging role in sculpting the tumour immune landscape and modulating immunotherapeutic responses. A critical appraisal of novel therapeutic strategies is also presented. Unresolved pathological mechanisms and translational gaps require further investigation and represent priority areas for future research.

## 2 Overview of autophagy

The autophagy process begins with the formation of phagophore; this is a crescent-shaped double-layer membrane structure, which will continue to extend and engulf cytoplasmic substances, and finally close to form autophagosome. Subsequently, this mature vesicle will fuse with the lysosome, and its internal substances will be degraded under the action of lysosome enzymes, thus realizing the recycling of nutrients (10).

**Figure 2:**
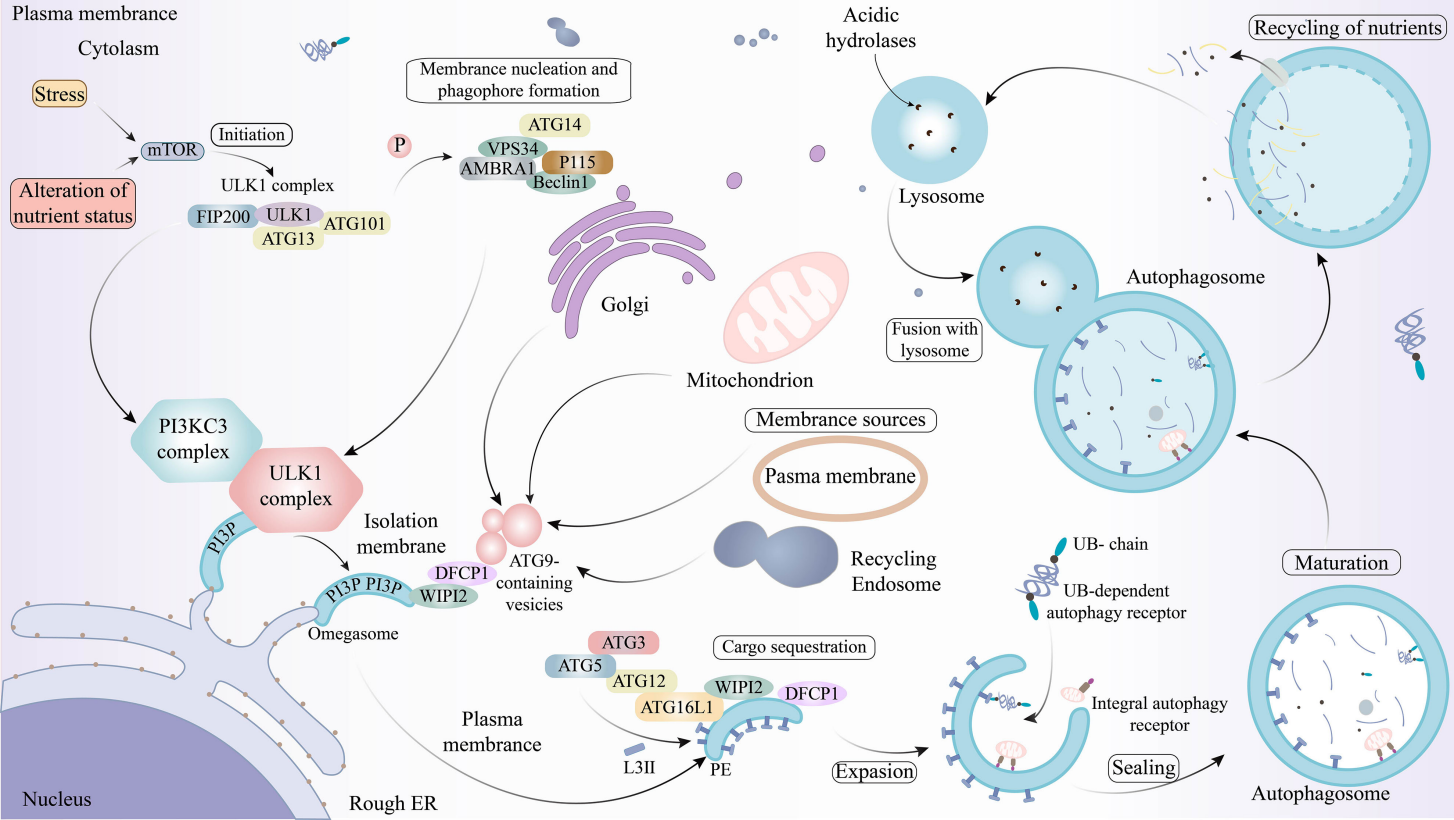
Autophagy core regulatory pathway. The diagram outlines macroautophagy, a sequential pathway comprising initiation, phagophore nucleation, vesicle expansion, lysosomal fusion, and cargo breakdown. Key regulatory modules include the ULK1 initiation complex, PI3KIIInucleation complex, ATG12 conjugation system and LC3 conjugation systems. (AMPK, AMP-activated protein kinase; Atg, autophagy-related gene; DFCP1, double FYVE-containing protein 1; ER, endoplasmic reticulum; FIP200, FAK family-interacting protein of 200 kDa; LC3, microtubule-associated protein 1A/1B-light chain 3; mTOR, mechanistic target of rapamycin; PI3KIII, class III phosphoinositide 3-kinase; ULK1, unc-51-like autophagy activating kinase 1; VPS15, vacuolar protein sorting 15; VPS34, vacuolar protein sorting 34; WIPIs, WD-repeat domain phosphoinositide-interacting proteins).

Autophagic flux represents a dynamic functional metric encompassing four sequential stages: substrate recognition and signalling, autophagosome assembly and substrate segregation, autophagosome–lysosome/late endosomal fusion and the release of enzymatic degradation products back to the cytoplasm (11) (**Figure 2**). This complete degradation pathway has been termed canonical autophagy.

The core of autophagy initiation lies in the regulatory balance between AMPK and the mammalian target of rapamycin complex 1 (mTORC1) (12, 13). Under nutrient-rich conditions, mTORC1 suppresses autophagosome formation by phosphorylating the ULK1 kinase complex, which comprises ULK1, ATG13, FIP200, and ATG101 (13). Conversely, during energy stress (such as ATP depletion), AMPK directly activates ULK1 and removes the inhibition mediated by mTORC1, thereby initiating the formation of autophagosomes (14).

Upon activation, the ULK1 complex recruits the autophagy-specific class III PI3K complex I (PI3KC3-C1), which contains Beclin1, to generate phosphatidylinositol 3-phosphate (PI3P) at the phagophore assembly site (15). This lipid signal is decoded by the PI3P-binding effectors WIPI2 and DFCP1, which are consequently recruited to the ER-derived omegasome. WIPI2 directly binds ATG16L1, recruiting the ATG12–ATG5–ATG16L1 complex, which acts as an E3-like enzyme to facilitate the ATG3-mediated conjugation of ATG8 family proteins (LC3 and GABARAPs) to membrane-resident phosphatidylethanolamine (PE), thereby generating lipidated, membrane-bound LC3-II (16, 17). This anchored LC3-II drives autophagosome elongation (18, 19). The ATG8 family proteins also coordinate the recruitment of LIR-containing autophagy factors and enable selective autophagy through interactions with cargo receptors (20).

Meanwhile, ATG9-containing vesicles act as essential lipid carriers, tethering the endoplasmic reticulum (ER) to nascent phagophores via ATG2A, thereby providing membrane scaffolds for phagophore nucleation and growth (21, 22). Membrane contact sites (MCSs) between the ER and the plasma membrane supply additional lipids (23). Once membrane sealing is complete, the autophagosome matures through ATG protein removal and fuses with lysosomes, where acidic hydrolases degrade the cargo and release nutrients for cellular reuse (24). This degradation and recycling step constitutes the final stage of the autophagic process.

Selective autophagy enables cells to specifically recognize and degrade particular substrates, such as mitochondria, ferritin, lipid droplets, and protein aggregates. This specificity is mediated by dedicated cargo receptors (e.g., p62/SQSTM1, NBR1, OPTN). A key feature of these receptors is their LC3-interacting region (LIR) motif, which directly binds to ATG8 family proteins (LC3/GABARAPs) anchored on the phagophore membrane, thereby tethering the cargo to the autophagic pathway.

Mitophagy is the selective clearance of damaged or superfluous mitochondria (25) and proceeds primarily through two major pathways. The ubiquitin-dependent pathway relies on the PINK1–Parkin axis, whereby loss of mitochondrial membrane potential leads to PINK1 stabilization on the outer mitochondrial membrane, recruitment and activation of the E3 ligase Parkin, and subsequent ubiquitination of mitochondrial proteins, which marks the organelle for recognition by ubiquitin-binding receptors such as p62 and OPTN (26–28). The receptor-mediated pathway utilizes mitochondrial outer membrane proteins containing LIR motifs, such as FUNDC1 and BNIP3/BNIP3L, that directly engage LC3 under specific conditions (29, 30).

Ferritinophagy is mediated by the cargo receptor NCOA4, which binds ferritin via its LIR motif and delivers it to autophagosomes for degradation, releasing free iron and thereby modulating intracellular iron homeostasis and redox balance (31). Aggrephagy relies on receptors such as p62 and NBR1, which recognize ubiquitinated protein aggregates and target them to autophagosomes for clearance, thereby alleviating proteotoxic stress (32). Lipophagy proceeds through mechanisms involving perilipin proteins (e.g., PLIN2 and PLIN3). These proteins, upon receiving corresponding signals, will expose LIR motifs or interact with soluble autophagy receptors, thereby leading to the selective degradation of lipid droplets and participating in the regulation of lipid metabolism (33).

Selective autophagy pathways are critically involved in gynaecological cancers. Li et al. reported that high FUNDC1 expression correlates with poorer overall and disease-free survival in cervical cancer, suggesting FUNDC1-mediated mitophagy as an independent prognostic factor. Mechanistically, FUNDC1 promotes hypoxia-induced mitophagy via direct LC3 interaction, and its dysregulation drives mitochondrial reprogramming to support cancer cell survival under stress. These findings indicate that targeting FUNDC1-mediated mitophagy may be a viable therapeutic strategy for cervical cancer (34). Similarly, ovarian cancer stem cells (CSCs) exhibit enhanced mitophagy driven by elevated BNIP3/BNIP3L expression. Hyperactivated NF-κB signalling, promoted by DNA-PK, upregulates these mitophagy receptors. Knockdown of BNIP3/BNIP3L or inhibition of NF-κB reduces mitophagy and impairs CSC self-renewal (35).

NCOA4-mediated ferritinophagy has been shown to promote ferroptosis and reverse cisplatin resistance in ovarian cancer, highlighting its therapeutic potential (36). In endometrial cancer, the expression of aggrephagy-related proteins is significantly associated with patient prognosis and the tumour microenvironment (37). Moreover, lipophagy can help gynaecological cancer cells resist chemotherapy by mobilizing stored lipids to provide energy for survival signals (38). However, the role of other subtypes of selective autophagy has not yet been fully investigated.

## 3 Autophagy and the tumour microenvironment

Autophagy plays a context-dependent dual role in gynaecological cancers. It suppresses tumour initiation but becomes indispensable for sustaining proliferation and metabolic activity in established neoplasms (39, 40).

In advanced ovarian cancer, the activity of autophagy signalling pathways is heightened; tumour cells utilise autophagy to gain a survival advantage in hypoxic and nutrient-deprived microenvironments, thereby developing resistance to chemotherapy and radiotherapy (41). In the context of high-risk human papillomavirus (HPV) infection and cervical carcinogenesis, autophagy exerts a protective function by limiting malignant transformation (42). Nevertheless, discussions regarding the pathological background of gynaecological cancers remain relatively scarce. The following section will explore the dual nature of autophagy within the tumour microenvironment, with a particular focus on the principles applicable to these female-specific malignancies.

### 3.1 Autophagy suppresses tumour development

As a fundamental cellular process, autophagy operates continuously to support macromolecular turnover, energetic equilibrium, and regulatory signalling (43). It is upregulated by stressors including nutrient deprivation, hypoxia, and infection, degrading cellular constituents to sustain metabolism (44). Through preserving cellular homeostasis, autophagy inhibits tumour development by preventing the buildup of damaged components under stressful conditions (45).

Early tumour genomic analyses revealed frequent hemizygous deletion of Beclin1 in breast and ovarian cancers. The tumour-prone phenotype of Beclin1-heterozygous mice offered initial genetic validation of the ability of autophagy to protect against cancer (46, 47). Further investigations have proposed that this deletion frequency is related to the chromosomal proximity of Beclin1 to the BRCA1 tumour suppressor at 17q21 (48).

Subsequent investigations have revealed that the tumour-suppressive functions of autophagy exhibit both tissue-specific and gene-specific characteristics. Following the knockdown of ATG5 to inhibit autophagy, OC stem cells completely lost their ability to self-renew, with the cell cycle arresting at the G0/G1 phase. The study further revealed that autophagy and NRF2 form a positive feedback loop, jointly regulating intracellular ROS levels, which in turn influences NOTCH1 expression. These findings highlight the critical role of the ATG5-mediated autophagy pathway in maintaining the characteristics of ovarian cancer stem cells (49).

The oncosuppressive functions of autophagy in most tissues typically depend on cooperative engagement with complementary molecular lesions. In cervical cancer (CC) cells, the knockdown of ATG5 leads to a decrease in migration and invasion abilities. Functional studies have shown that the knockdown of ATG5 can reverse the epithelial–mesenchymal transition (EMT) process through pathways such as P-ERK, P-NFkBp65, and P-mTOR (50).

Furthermore, autophagy itself is subject to regulation by tumour-suppressive pathways, most notably through a complex, bidirectional regulatory relationship with the p53 protein. Under homeostatic conditions, cytoplasmic p53 suppresses autophagic activity, whereas under stress conditions such as DNA damage, activated p53 transcriptionally upregulates the expression of proautophagic genes, including DRAM1 (51, 52). Conversely, autophagy can reciprocally modulate p53 activity through both suppression of its activation and chaperone-mediated degradation of mutant p53 (53, 54). This intricate bidirectional regulatory network further reinforces the active tumour-suppressive role of autophagy.

In addition to bulk autophagy, selective autophagy subtypes are critical for removing damaged organelles and preventing oncogenic stress. Mitophagy eliminates dysfunctional mitochondria, and the loss of mitophagy genes leads to the accumulation of defective organelles, triggering ROS bursts and DNA damage that directly drive tumorigenesis. For example, in endometrial cancer, elevated BNIP3 expression correlates with active HIF-1α signalling and poor prognosis, indicating its involvement in disease progression (55). Moreover, BNIP3 is upregulated in type I endometrial carcinoma tissues compared to matched non−malignant tissues, suggesting an adaptive response to mitochondrial dysfunction in cancer cells (56).

Studies have shown that the ubiquitination of GAPDH is essential for driving mitophagy, and activating this pathway can significantly inhibit tumour growth. On the contrary, when GAPDH undergoes mutations and cannot be ubiquitinated, the glycolysis of CC cells significantly increases, and the tumour-forming ability in the body also significantly improves (57). Pexophagy, which clears peroxisomes, also modulates the ROS balance; although its role in cancer is less defined than that of mitophagy, it may be crucial given the importance of fatty acid β-oxidation in tumorigenesis (58, 59).

Additionally, in autophagy-deficient mouse models of cancer, simultaneous deletion of p62 reverses tumorigenesis (60), indicating a dual role for p62 in carcinogenesis. When p62 accumulates owing to defective autophagy, it aberrantly activates the pro-oncogenic NF-κB and NRF2 signalling pathways.

### 3.2 Autophagy promotes tumour development

Early observations revealed an increased abundance of LC3-positive puncta and lipidated LC3-II in specific malignancies, suggesting autophagosome accumulation (61). Nevertheless, these static histological assessments merely quantify vesicle numbers and cannot distinguish whether they reflect increased autophagosome formation or impaired lysosomal clearance.

Research using carcinogenic RAS-driven tumour models has demonstrated the autophagy dependency in certain cancers. In gynaecologic oncology, RAS mutations exhibit a distinct histopathologic distribution and may impact overall survival (62). For instance, in KRAS-mutant ovarian cancer models, the autophagy-mediating protein DIRAS3 has been shown to induce autophagic flux and confer adaptive resistance to chemotherapy, highlighting the dependency of RAS-driven gynaecologic tumours on autophagic pathways (63).

Beyond the influence of oncogenes, tumour development requires coordinated interactions with tumour suppressor pathways. In patients with endometrial cancer, the expression levels of the ATG4B gene and protein in endometrial cancer tissues are elevated, indicating that it has a role in promoting tumour development (64). In ovarian cancer, the LKB1-AMPK axis orchestrates metabolic stress responses and is critical for metastasis. Intact LKB1 activity sustains dormant cancer spheroids, key intermediates of peritoneal spread. LKB1 can also promote metastasis via AMPK-independent pathways, and its dysfunction correlates with metabolic adaptation of tumour cells under stress (65, 66).

Furthermore, autophagy promotes tumour progression by modulating the metabolic environment to support cancer cell proliferation (67). In tumour regions that experience metabolic stress from hypoxia and nutrient deficiency, autophagy increases cell viability through metabolite generation and oxidative stress management (68). Hypoxic conditions activate HIF-1α and BNIP3 signalling, which competitively disrupts the association of Beclin1 with BCL-2 to launch a protective autophagy program that enhances malignant cell adaptation to oxygen limitation (69, 70). However, the crucial point is that prolonged or severe hypoxia can also trigger autophagy-dependent cell death in certain cases, indicating that the cell protection function of HIF-1α-driven autophagy depends on the specific environment, and the threshold that distinguishes survival from death is still unclear.

In ovarian cancer, Rab11a expression is upregulated; its knockdown inhibits cancer cell proliferation, migration and invasion, and induces cell cycle arrest. The autophagy inhibitor 3-MA reverses the promoting effects of Rab11a overexpression on the aforementioned malignant phenotypes and autophagy, suggesting that Rab11a drives the progression of OC by regulating the autophagy pathway (71). Furthermore, in gynaecological cancers such as cervical and ovarian cancer, the transferrin receptor (TFRC) is highly expressed. Both iron accumulation and autophagy can promote tumour growth, whilst the activation of autophagy can regulate TFRC expression (72, 73).

Research further demonstrates that neoplastic cells metabolically reprogram themselves under hypoxia, transitioning from oxidative phosphorylation toward glycolytic flux while channelling resulting intermediates into anabolic processes like the pentose phosphate pathway to support nucleotide production (74). This metabolic reprogramming necessitates precise regulation of mitochondrial quantity, as certain anabolic reactions still require functional mitochondria. Selective autophagy, particularly mitophagy, eliminates dysfunctional mitochondria to prevent their accumulation and subsequent excessive ROS production (75). Nevertheless, excessive mitophagy may deplete the total mitochondrial pool required for lipid and nucleotide biosynthesis, suggesting that there is an optimal threshold for mitophagy, which may vary depending on the type of cancer and its stage of progression.

Scientific research has confirmed that the dysregulation of mitochondrial autophagy in ovarian cancer leads to chemotherapy resistance and disease progression. For instance, inhibiting mitochondrial autophagy mediated by BNIP3 can reduce the resistance of ovarian cancer cells to cisplatin (76). Moreover, in the ovarian cancer peritoneal metastasis model, it was found that a portion of TAMs with higher mitochondrial autophagy function can promote tumour growth *in vivo (*77).

The ROS levels frequently observed in autophagy-deficient cells can be alleviated through the antioxidant transcriptional process mediated by the NRF2 activated by p62 accumulation. In ovarian cancer, the highly expressed p62 in cisplatin-resistant cells protects cancer cells from oxidative damage by competing with NRF2 for binding with Keap1, thereby promoting NRF2 activation and chemotherapy resistance. Additionally, p62 also plays an important role in cell apoptosis and autophagy by integrating the Keap1-NRF2 and NF-κB signalling pathways, promoting tumour progression and treatment resistance (78).

To investigate the precise mechanisms by which autophagy inhibition enhances tumour cell metastasis, researchers have conducted more in-depth investigations of these findings. The aggregation of selective autophagy receptors such as p62 drives neoplastic advancement and treatment resistance in cells with impaired autophagy via the activation of multiple signalling cascades (79).

Empirical data confirm that the accumulation of p62 can stabilize the epithelial–mesenchymal transformation regulator TWIST1, thus enhancing the metastatic process. TWIST1 is a well-established driver of metastasis in ovarian cancer, promoting mesothelial clearance and invasion (80). Moreover, in endometrial cancer, high cytoplasmic p62 expression correlates with deep myometrial invasion, vascular invasion, and poor prognosis. Conversely, p62 knockdown reduces cancer cell invasiveness and tumour growth *in vivo*, likely through NF-κB activation (81).

Empirical data confirm that the accumulation of p62 can stabilize the epithelial–mesenchymal transformation regulator TWIST1, thus enhancing the transfer process in living organisms (82). For example, in ovarian cancer cells, p62-mediated activation of the NF-κB pathway (which is partially dependent on RIP1) promotes cisplatin resistance, cell proliferation and the inhibition of apoptosis, thereby exacerbating malignant progression. Similarly, in cervical cancer, studies have shown that autophagy regulates cell invasiveness via the NF-κB pathway by modulating p62 levels in the cytoplasm and nucleus (83).

**Figure 3:**
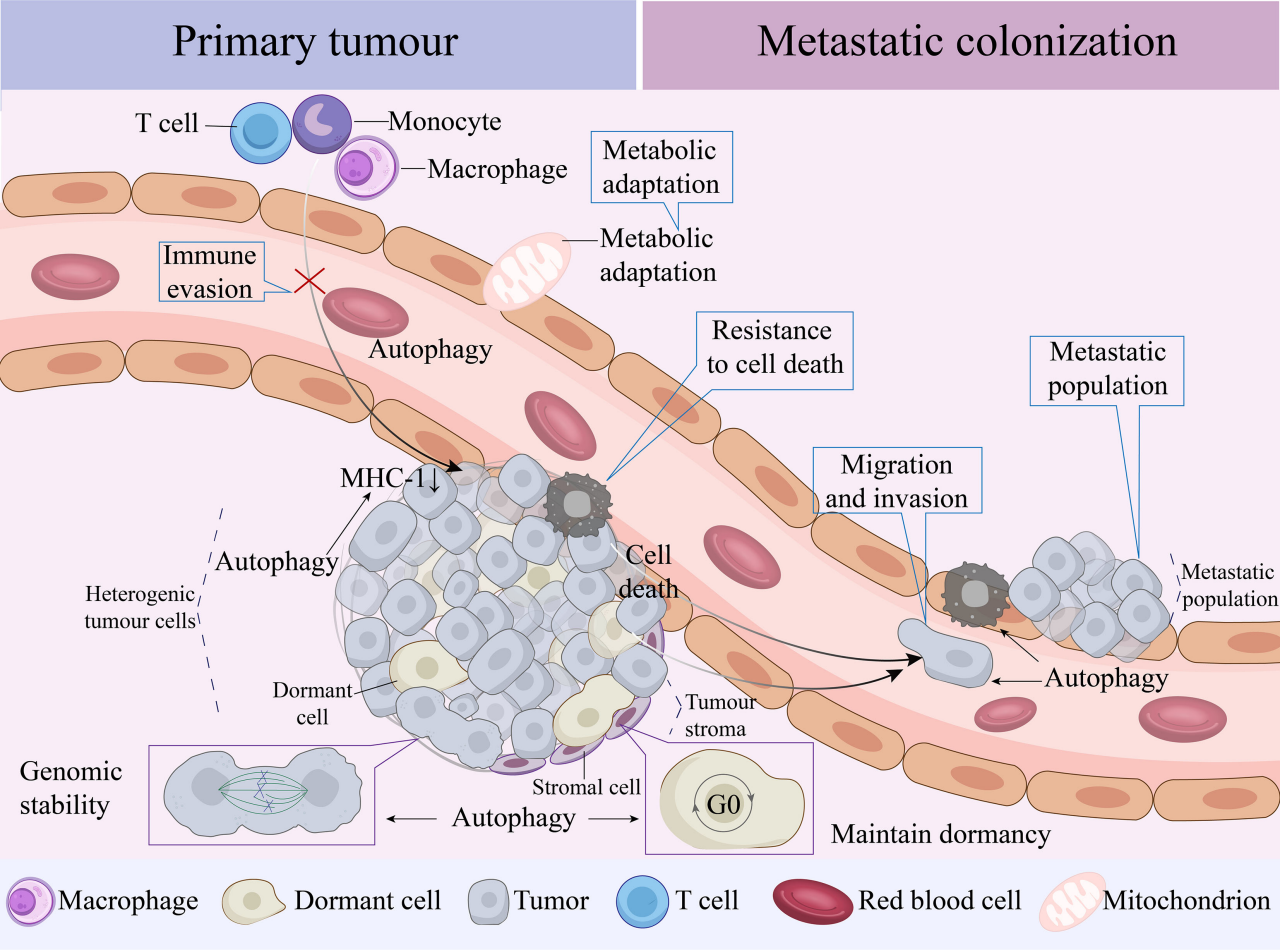
The role of autophagy in primary tumours and metastasis. Autophagy supports tumour growth and survival through multiple mechanisms. In terms of tumour promotion, autophagy plays a critical role in regulating metabolic adaptation of tumour cells and facilitating immune evasion. In contrast, its function in metastasis is more complex. Autophagy enables tumour cells detached from the extracellular matrix to resist detachment-induced cell death. Simultaneously, it helps maintain tumour cell dormancy and preserves genomic stability. (G0, gap 0phase; MHC-I, major histocompatibility Complex class I).

A subset of proteins and genetic loci that fuel tumour growth, p62 being a paradigmatic example, partially overlaps with those implicated in tumour suppression, indicating that autophagy exerts qualitatively distinct regulatory effects across different cancer types and at successive stages of metastasis. Future mechanistic research must focus on chamber-specific autophagy functions related to the cancer spectrum that drive the mutation and dissemination stage to clarify the underlying regulatory role of autophagy in the entire process of tumour development (**Figure 3**).

### 3.3 Autophagy in the immune microenvironment

Regulation of the immune microenvironment plays a crucial role in shaping tumour development and malignant progression, thus establishing immunotherapy as the cornerstone of treatment in the field of oncology (8). Autophagy can exert immune regulatory effects that vary depending on the specific circumstances. The details are as follows.

#### 3.3.1 Effect of autophagy on PD-L1, CTLA-4, and MHC expression

Notably, programmed death-ligand 1 (PD-L1) constitutes a critical immune checkpoint molecule within the tumour microenvironment. It is expressed on various cell types including malignant cells, macrophages, and dendritic cells. Binding of PD-L1 to its receptor PD-1 on T lymphocytes inhibits T cell effector functions, consequently inducing T cell deletion, functional attenuation, or exhausted states that collectively promote tumour immune escape (84, 85). Conversely, autophagy activation generally promotes the degradation of PD-L1, resulting in its downregulation and subsequently enhancing anti-tumour immunity. Its net effect on tumour immunity is highly dependent on the specific tumour microenvironment.

The most direct mechanism identifies PD-L1 as a candidate substrate for autophagy degradation, and uses cargo receptors such as p62 to direct it to lysosomes. In ovarian cancer and melanoma models, PD-L1 directly drives tumour progression by inhibiting autophagy and activating mTORC1, and knocking down PD-L1 can inhibit tumour growth and metastasis in immunodeficient mice (86). Mechanism studies have found that usnic acid can induce the nuclear translocation of the MiT/TFE family by inhibiting the mTOR pathway, promote lysosome formation, and thereby accelerate the lysosomal degradation of PD-L1 (87).

At the molecular level, TRIM14 recruits the deubiquitinase USP14 to stabilize PD-L1 by removing its K63-linked ubiquitin chains, thereby preventing its degradation by p62-mediated autophagy (88). In cervical cancer, inhibition of USP14 has been shown to promote MDM2 degradation and induce tumour cell apoptosis (89). In ovarian cancer, the expression of USP14 mRNA is found to be related to tumour grade. Knockdown of USP14 can alleviate malignant characteristics and restore the sensitivity of drug-resistant cells to cisplatin. Pharmacological inhibition of USP14 using the small molecule inhibitor ARN12502 can induce apoptosis in cisplatin-resistant ovarian cancer models (90).

Autophagy and related signalling pathways also indirectly regulate PD-L1 expression. For instance, in ovarian cancer, the tumour suppressor gene ARHI (DIRAS3) can inhibit the PI3K/AKT and Ras/MAP signalling pathways, reduce the phosphorylation of FOXO3a, promote its nuclear translocation, and thereby activate the transcription of autophagy-related gene Rab7, driving autophagy to occur (91).

However, research has also shown that the activation of autophagy can promote the stabilisation of the immune microenvironment via PD-L1. Gupta et al. demonstrated that PD-L1 attenuation in murine ovarian cancer cells augments autophagy and blunts tumour growth, while anti-PD-L1 antibody treatment exhibits tumour-specific effects on growth and metastasis in NSG mice, illustrating that PD-L1 exerts tumour cell-intrinsic signals critical for OC pathogenesis (86). Therefore, bidirectionally modulating autophagic activity to maintain PD-L1 homeostasis holds significant implications for therapeutic intervention.

Further clinical studies have shown that CAMKKβ, as the key upstream kinase of AMPK, when its gene is knocked out, it significantly inhibits the phosphorylation of AMPK (at the Thr172 site) and its downstream effector molecule ULK1 (at the Ser555 site) in epithelial ovarian cancer (EOC) spheres, thereby weakening the autophagic flux and reducing the survival and metastasis potential of cancer cells (92). Thus, targeting the AMPK-ULK1 axis may represent a strategy to modulate the immune microenvironment in ovarian cancer.

In contrast, therapeutic blockade of autophagy upregulates PD-L1 presentation. Cinchonine enhances PD-L1 expression in lung cancer models by suppressing autophagic degradation, and its combination with anti-PD-L1 agents markedly inhibits tumour progression (93). Thus, both inducing and suppressing autophagy can modulate PD-L1 levels and anti-tumour immunity, but the effect is highly drug- and context-specific. Without detailed molecular validation, this regulation cannot be applied as a universal therapeutic strategy.

The immunosuppressive molecule CTLA-4, highly expressed on Tregs, is also subject to degradation by the autophagy−lysosomal system, thereby finely tuning the inhibitory capacity of these cells (94, 95). Inhibition of autophagy (for example, through the use of chloroquine) can stabilize CTLA-4 and may enhance its immunosuppressive effect, suggesting that autophagy inducers may have a synergistic effect with CTLA-4 blocking therapy, thus bringing therapeutic benefits (94, 96). This regulatory network extends to dendritic cells. Foxp3^+^ Tregs enhance PI3K/AKT/mTOR signal conduction in dendritic cells and weaken their autophagy activity through the mechanism mediated by CTLA-4, thus reducing their antigen processing ability and reducing autoimmune pathological damage in the body (97).

Major histocompatibility complex (MHC) molecules, alternatively termed human leukocyte antigens, function as cell surface receptors on antigen-presenting cells that capture and display antigenic fragments to T lymphocytes, consequently activating and modulating adaptive immunity. Among these, MHC class I (MHC-I) is a membrane protein complex expressed on the surface of nearly all nucleated cells. Its core function is to present endogenous antigenic peptides to CD8^+^ cytotoxic T lymphocytes (CTLs), thereby initiating or sustaining cellular immune responses such as antiviral and antitumour immune responses (98).

In the mouse ovarian cancer model, it was found that the expression of XPR1 was significantly upregulated. This was achieved through interaction with LAMP1 and by controlling the localization and synthesis of MHC-I molecules on the surface of OC cells via the autophagy pathway, thereby promoting the growth and immune evasion of ovarian cancer (99). Additionally, studies have shown that the autophagy-dependent MHC-I quality control mechanism mediated by IRGQ is an important driver of pan-cancer immune evasion, and its regulatory mechanism is applicable to various epithelial solid tumours including ovarian cancer. Specifically, the molecular cargo receptor IRGQ directs misfolded MHC-I complexes toward lysosome-dependent elimination through associations with autophagy adaptors GABARAPL2 and LC3-II, representing a form of selective autophagy (100).

Mechanistically, the autophagy cargo receptor NBR1 directly interacts with MHC-I molecules to mediate their incorporation into autophagic structures destined for lysosomal breakdown (101). This process is precisely regulated by multiple factors. For instance, in endometrial cancer, the autophagy protein MAP1LC3/LC3 can interact with the trans-activating factor NLRC5 of MHC-I, inhibiting the MHC-I gene expression and antigen presentation pathway mediated by NLRC5 (102). Additionally, in the study of cervical cancer, IFN-γ treatment significantly induces the autophagy process in HeLa and SiHa cells by upregulating the expression of IDO1 and increasing tryptophan accumulation, thereby promoting the phagocytosis and activation of macrophages (103).

However, under specific conditions, autophagy can also upregulate MHC-I expression. For instance, during radiation therapy (at single doses ranging from 2 to 20 Gy), induced autophagy increases MHC-I expression in a dose-dependent manner, which is correlated with enhanced CD8^+^ T-cell infiltration (104).

This indicates that autophagy may promote antigen presentation by clearing the intracellular environment or influencing signalling pathways. The regulatory process in this case is influenced by the cytokine microenvironment. In ovarian cancer, treatment with IFN-γ and TNF-α increases MHC-I expression in all tested ovarian tumour cell lines and alters their sensitivity to NK cell-mediated cytotoxicity (105).

Extending this perspective to clinical practice, IFN-γ levels determine the direction of autophagy regulation on MHC-I. In HPV-positive cervical cancer, immune checkpoint inhibitors (ICBs) induce IFN-γ-mediated reorientation of autophagy to enhance antigen presentation (106). This framework provides a mechanistic basis for stratifying gynaecological cancer patients based on tumour IFN-γ signatures, with a view to implementing autophagy-modulating combination immunotherapy. However, the direct regulation of other immune checkpoints by autophagy remains unexplored in gynaecological malignancies and warrants future investigation.

#### 3.3.2 Autophagy in T cells

In addition to playing a role in immune molecular regulation, autophagy also constitutes a basic process that is responsible for coordinating the activities of tumour-infiltrating immune cells such as T lymphocytes, macrophages, and myeloid-derived suppressor cells. By regulating proliferation, metabolic fitness, differentiation programs and survival pathways, autophagy widely affects the intensity and quality of antitumour immune responses (107, 108).

Although T cells represent crucial mediators of antitumor immunity, experimental evidence demonstrates that autophagy impairment promotes their apoptotic death and restricts population expansion, consequently weakening the overall T cell response (109). Specifically, autophagy defects will disrupt the TCR-mediated CDKN1B (a key regulatory factor in the T cell cycle process) degradation process, causing CD8^+^ T cells to arrest in the G1 phase, thus unable to start DNA replication (110).

In ovarian cancer, malignant ascites frequently accumulates within the abdominal cavity, thereby creating a unique immunosuppressive microenvironment (111). Oxidised cholesterol in the ascites disrupts cholesterol homeostasis within T cells by simultaneously inhibiting the SREBP2 transcriptional pathway and stimulating LXR signalling, leading to impaired T-cell proliferation and autophagy-mediated apoptosis, which in turn results in T-cell exhaustion and dysfunction (112).

In addition, studies in ovarian cancer patients and tumour-bearing mice reveal that tumour-derived factors such as lactate and oxysterol inhibit FIP200 expression, leading to autophagy dysfunction in naïve T cells. This dysfunction causes mitochondrial hyperactivation and increased ROS production, which in turn promotes T cell apoptosis and facilitates immune escape (113). Thus, the disturbed tumour microenvironment indirectly compromises T cell survival through the autophagic pathway.

Beyond controlling cellular division and survival, autophagy modulates T lymphocyte differentiation and functional specialization. Research has verified that genetic deletion of ATG5 or ATG7 substantially compromises the generation of memory T cells but minimally affects the immediate responsiveness of effector T cells. During ATG5 deficiency, T lymphocytes display a pronounced bias towards effector memory differentiation, resulting in elevated quantities of IFN-γ and TNF-α that may cooperatively enhance both inflammatory reactions and antitumour immunity (109, 114). Consequently, autophagy defects weaken long−lasting immune memory while potentially increasing short−term effector cytokine production. The net effect on tumour control thus depends on the malignancy type and treatment stage, underscoring the need for continuous immune monitoring.

The suppression of autophagy due to ATG5 deficiency triggers metabolic reprogramming in CD8^+^ T cells, potentially providing a transient increase in their tumoricidal capacity (115). Nevertheless, persistent defects in mitochondrial clearance ultimately result in the buildup of damaged organelles, driving T lymphocytes towards functional exhaustion over time.

Autophagy also influences the differentiation of helper T cell subsets. Deletion of PIK3C3/VPS34 reduces mitochondrial activity in activated T cells, impairing CD4^+^ T cell glycolysis and inhibiting their differentiation into Th1 cells (116). CD4^+^ T cells lacking ATG3 or ATG5 exhibit selective blockade of Th9 differentiation due to impaired autophagy, which paradoxically enhances the antitumor function of Th9 cells (117).

Autophagy plays an indispensable role in maintaining the functional integrity and immunosuppressive ability of regulatory T cells (Tregs), because its damage can lead to dysfunction of Tregs (118, 119). Mechanistically, autophagy deficiency in Tregs increases upstream signalling, including mTORC1 activation, amplifies MYC transcriptional activity, and accelerates glycolytic metabolism, collectively driving Treg dysfunction (119). The mTORC1/C2 inhibitor AZD8055 further suppresses autophagic processes and intensifies mitochondrial stress in Tregs, resulting in mitochondrial impairment, restricted Treg expansion, and diminished immunosuppressive potential (120). In ovarian cancer, where Treg infiltration is associated with poor prognosis, these findings suggest that targeting dysfunctional autophagic Tregs may offer a therapeutic opportunity (121).

#### 3.3.3 Autophagy in other immune cells

Antigen presentation is a core process in the regulation of the immune microenvironment, mainly carried out by dendritic cells (DCs) and macrophages. Among them, DCs are the cells with the strongest antigen-presenting ability, and autophagy has a key impact on antigen processing and presentation. On the one hand, during the differentiation of DCs, treatment with IL-4 regulates the mTORC1 signalling pathway and upregulates RUFY4, thereby inducing autophagic flux, promoting DC maturation, MHC-II expression, and T cell activation capacity (122).

On the other hand, LC3-II can promote the endocytosis and autophagic degradation of MHC-I molecules, thereby weakening T cell activation. Conversely, knockout of ATG5 or ATG7 disrupts the AAK1-MHC-I interaction, thereby impairing the internalization of MHC-I (123). These contradictory observations indicate that autophagy has context-dependent effects on antigen presentation, possibly promoting MHC-II-restricted responses while limiting the cross-presentation of certain antigens. In fact, autophagy seems to preferentially affect DC subsets specifically responsible for the cross-presentation of soluble protein antigens, while having minimal impact on cell-associated antigens or antigens taken up through receptor-mediated pathways (124).

Similarly, macrophages, another key type of phagocytic and antigen-presenting cells (125), exhibit a phenotypic dichotomy in the tumour microenvironment: the inflammatory M1 subtype with anti-tumour properties and the alternatively activated M2 subtype that supports tumour progression (126).

Studies have shown that inhibiting the autophagic activity of macrophages can induce a phenotypic shift towards M2 polarization, thereby promoting malignant progression. For instance, in CC patients, HPV16 E6 induces M2-type macrophages to polarize in the cervical microenvironment through exosomal miR-204-5p. The exosome pathway is closely related to the autophagy pathway in the generation and secretion of extracellular vesicles (127). In endometrial cancer, the high glycolysis of tumour cells produces a large amount of lactic acid, which regulates the autophagic flux through the modulation of AMPK/mTOR pathways to drive the M2 polarization of tumour-associated macrophages (TAMs), and promotes tumour progression by secreting factors such as IL-6 (128).

Furthermore, mTOR activation and TRAF2 stabilization are among the pathways that drive this phenotypic shift, enabling TAMs to perform supportive functions such as promoting tumour angiogenesis (129, 130). RNF126 facilitates PTEN ubiquitination, inducing PI3K/AKT pathway activation and autophagy restriction. The resulting signalling enhancement increases macrophage migratory capacity (131).

Conversely, upregulation of NOD1 or NOD2 in cervical squamous cell carcinoma (CSCC) promotes proliferation, invasion, and migration through activation of NF-κB and ERK signalling pathways, leading to poor prognosis (132). Cryptotanshinone acts as an antitumor immunomodulator that converts tumour-associated macrophages (TAMs) from the M2 to the M1 phenotype, leading to tumour regression (133). Additionally, it suppresses ovarian cancer cell proliferation, migration, and invasion by promoting ubiquitination and degradation of c-Myc, disrupting the c-Myc/Max interaction, and attenuating the FAK signalling pathway, thereby enhancing sensitivity to paclitaxel and cisplatin and inhibiting orthotopic tumour growth (134).

Accordingly, asparaginase activates the AKT/mTOR signalling pathway while inhibiting the ERK1/2 pathway to block autophagy, thereby weakening phagocytic activity, cytokine production, and MHC-II expression in macrophages (135). CSF-1-induced differentiation of monocytes into macrophages is an autophagy-dependent process; inhibiting autophagy effectively blocks differentiation, suggesting a potential therapeutic strategy for depleting macrophages in cancer treatment (136).

In contrast, ER stress induces elevated CD244 expression in monocytes, whereas genetic ablation of CD244 stimulates autophagic activity in these cells and facilitates their differentiation into tumour-suppressive macrophages (136). Thus, autophagy exerts context-dependent regulatory functions in macrophages, either promoting or suppressing tumour immunity depending on the cellular state.

In addition to the classic antigen-presenting cells, myeloid-derived suppressor cells (MDSCs) suppress T cell activation and proliferation, thereby blocking the initiation of anti-tumour immune responses. MDSCs include monocyte subtypes (M-MDSC) and polymorphonuclear cell subtypes (PMN-MDSC) (137). These cells significantly contribute to malignant advancement and dissemination, with autophagic processes fundamentally sustaining the immunosuppressive capacity of M-MDSCs. Autophagy deficiency leads to upregulated MHC-II expression on M-MDSCs, thereby attenuating their suppressive capacity (138). Signalling mediators including HMGB1 and β2-AR, maintain MDSC viability through autophagy activation and stimulate the production of immunosuppressive factors such as PGE2, collectively accelerating malignant progression (139).

As innate lymphoid cells, natural killer (NK) cells not only mediate direct anti-tumour and antiviral activity, but also play the role of immunomodulator (140). The ovarian cancer microenvironment secretes CXCL12, which interacts with the overexpressed CXCR4 receptor on tumour-infiltrating NK cells, thereby blocking the autophagic flux and inhibiting the function of NK cells. CXCR4 antagonists can reverse this immunosuppression, enabling NK cells and CAR-NK cells to effectively control tumour growth (141).

Furthermore, phosphatidylcholine is a key immunosuppressive metabolite in the ascites of high-grade serous ovarian cancer (HGSOC), and its uptake mediated by SR-B1 disrupts the orderliness of NK cell membranes and their cytotoxic functions. Blocking this lipid uptake pathway can restore the anti-tumour activity of NK cells, providing a new target for overcoming immune suppression mediated by ascites (142).

Mechanistically, autophagy impairment in NK cells causes mitochondrial damage, elevates ROS levels, and reduces ATP production. These dysfunctions promote NK cell apoptosis, impair their migration, and decrease secretion of key cytokines such as IFN-γ, ultimately compromising antitumor efficacy (143, 144). These metabolic defects resemble those observed in autophagy-deficient T cells, suggesting conserved immunometabolic dysregulation pathways across lymphocyte lineages. However, direct comparative evidence across immune cell subtypes remains lacking.

**Figure 4:**
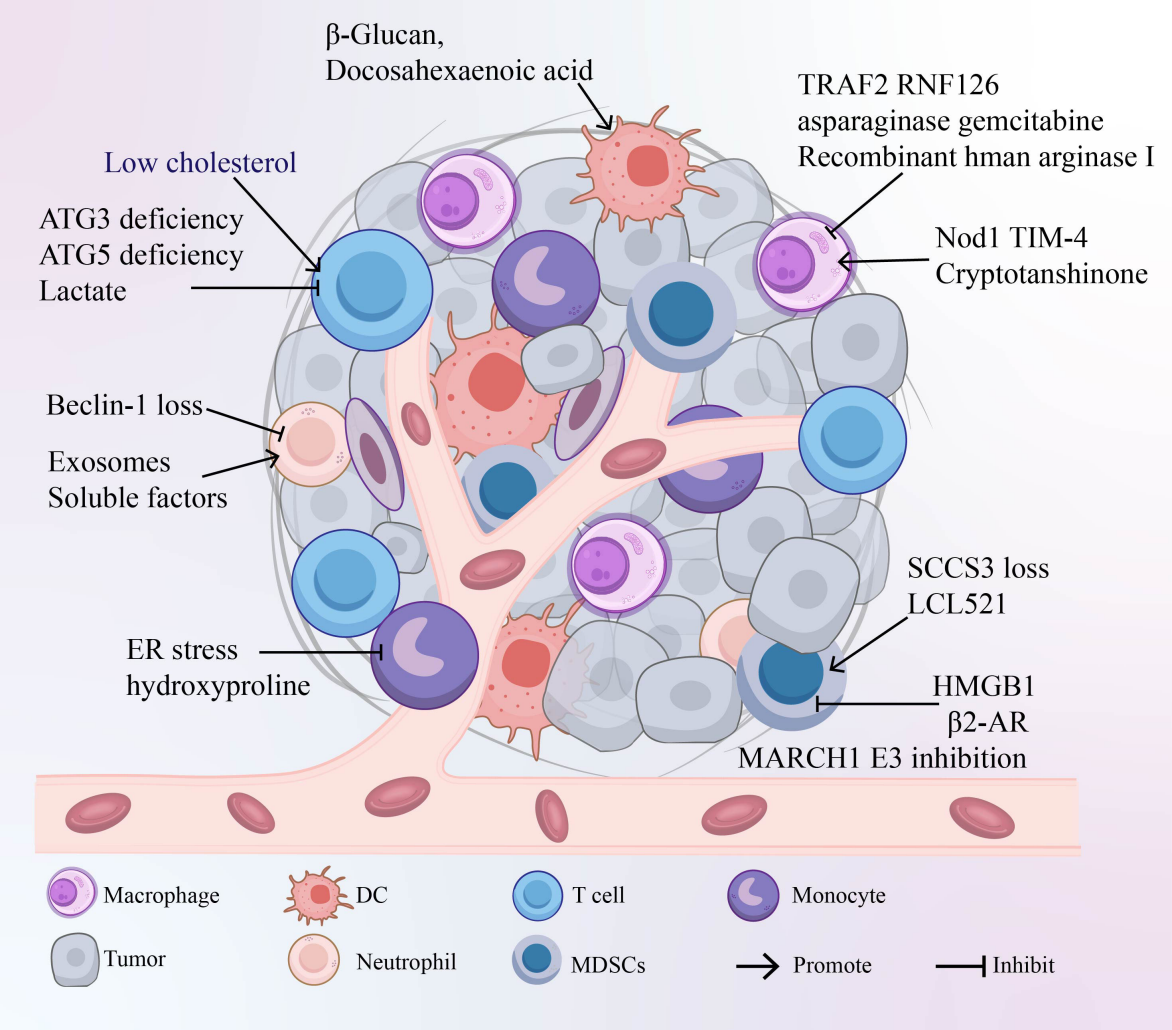
Autophagy in immune cells. Autophagy exerts complex and multifaceted regulatory functions in various immune cells within the tumour microenvironment, including T cells, macrophages, monocytes, dendritic cells, neutrophils, and myeloid-derived suppressor cells (MDSCs). In the context of gynaecological cancers such as ovarian and cervical cancer, autophagy significantly influences the function of key immune cell populations. For example, endoplasmic reticulum stress and hydroxyproline can inhibit autophagy in monocytes, thereby exerting immunosuppressive effects. Conversely, stimulatory agents such as β-glucan and docosahexaenoic acid promote autophagy in dendritic cells, leading to immune activation. Key regulatory molecules (e.g., TRAF2, RNF126, Nod1) are indicated in the image. These regulatory mechanisms highlight the intricate crosstalk between autophagy and immunity, offering potential novel therapeutic strategies for gynaecological cancers. (Atg, autophagy-related gene; DC, dendritic cells; E3, E3 ubiquitin ligase; ER, endoplasmic reticulum; HMGB1, high mobility group box 1; MARCH1, membrane-associated RING-CH-type protein 1; MDSC, myeloid-derived suppressor cells; Nod1, nucleotide-binding oligomerization domain-containing protein 1; RNF126, Ring Finger protein 126; TIM-4, T cell immunoglobulin and mucin domain-containing protein; TRAF2, TNF Receptor-Associated Factor 2).

In general, autophagy has an indispensable influence on the reshaping of the tumour microenvironment (**Figure 4**). Systematic analysis of its situation-dependent functions and molecular regulatory networks in tumour cell groups and immune cell groups will help to explore feasible treatment targets, thus providing a new conceptual framework for enhancing anti-tumour immunity and optimizing immunotherapy solutions.

## 4 Autophagy in female reproductive system tumours

The benign and malignant proliferations in the female reproductive organs are systematically classified according to their structural positioning and tissue morphology. Contemporary research has established that autophagy is a fundamental determinant of disease progression, which can activate complex signal transduction networks.

### 4.1 Endometrial cancer

Endometrial carcinoma (EC), an epithelial malignancy originating in the uterine lining, is clinically categorized as either type I (oestrogen-driven endometrioid) or type II (oestrogen-independent) (145). Type II includes serous papillary carcinoma, clear cell carcinoma, adenosquamous carcinoma, and mucinous carcinoma (146).

**Figure 5:**
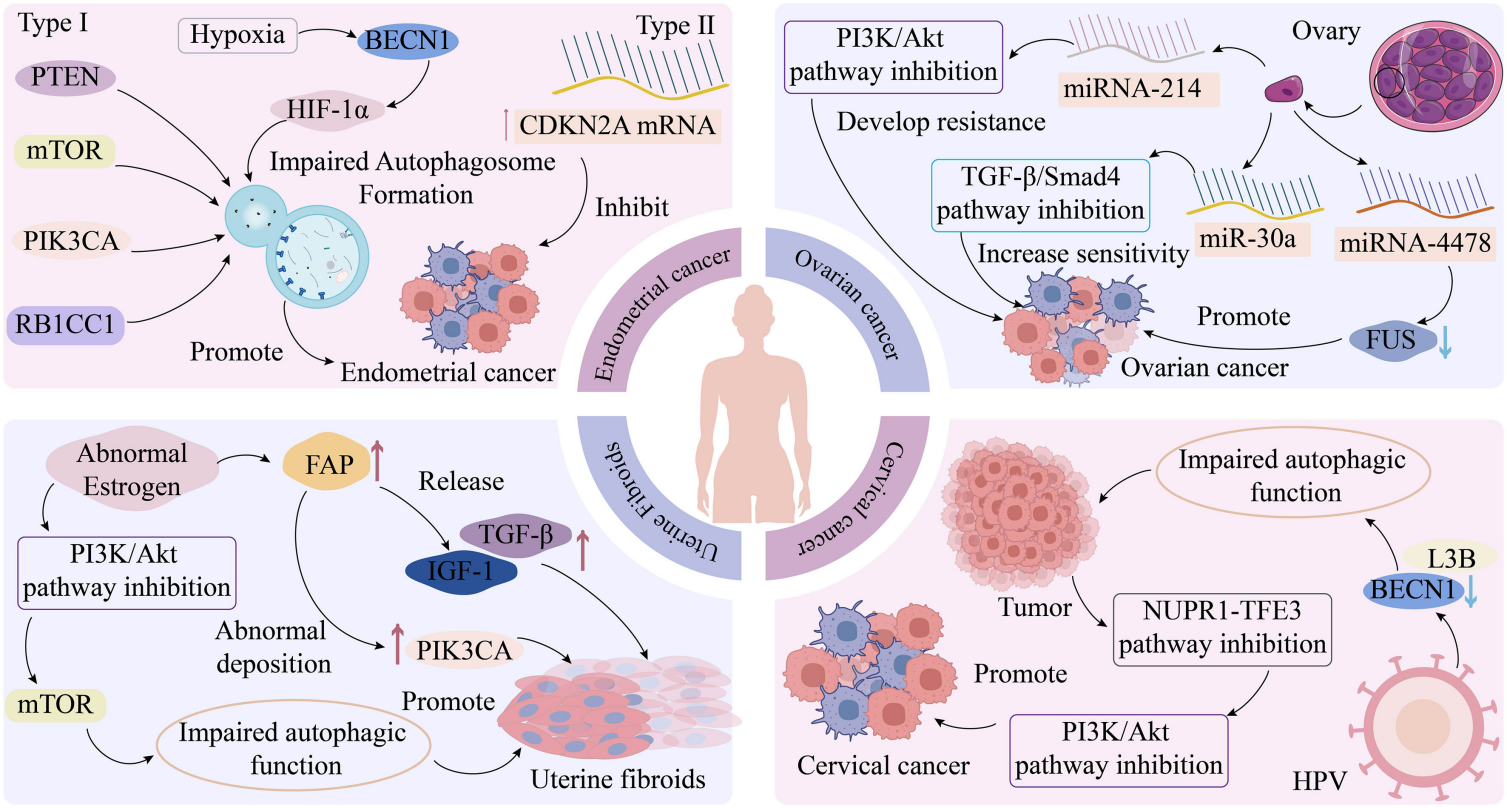
Autophagy in female reproductive system tumour. This schematic delineates pathogenic mechanisms of autophagy dysregulation in female reproductive tumours (ovarian cancer, cervical cancer, endometrial cancers and uterine fibroids. The diagram succinctly outlines relevant evidence supporting how autophagy and related processes contribute to the development of reproductive system tumours. (Akt, Ak strain transforming; CDKN2A, cyclin, dependent kinase inhibitor 2A; FAP, fibroblast activation protein; FUS, fused in sarcoma; HER2, human epidermal growth factor receptor 2; HIF, 1α, hypoxia, inducible factor 1, alpha; IGF, 1, insulin, like growth factor 1; miRNA, microRNA; mTOR, mechanistic target of rapamycin; NUPR1, nuclear protein 1; PI3K, phosphoinositide 3, kinase; PTEN, phosphatase and tensin homolog; RB1CC1, RB1, inducible coiled, coil protein 1; TFE3, transcription factor E3; TGF, β, transforming growth factor, beta).

Autophagy, a core regulator of cellular metabolic homeostasis, critically regulates endometrial cancer pathogenesis via pathways including metabolic reprogramming, signalling dysregulation, and microenvironment remodelling (147) (**Figure 5**). Genomic data from The Cancer Genome Atlas (TCGA) have demonstrated that the greatest prevalence of mutations in autophagy-regulating genes is among solid EC tumours, with the highest incidence in oestrogen-dependent (type I) cases (148).

Mutations in core autophagy genes (ATG4C, ULK4, and RB1CC1) and activation of the MTOR pathway are the main genetic features. Notably, dual mutations in MTOR and PTEN as well as loss-of-function mutations in RB1CC1 can lead to impaired autophagosome formation, suggesting that impaired autophagic processes may promote endometrioid cancer progression (149). In contrast, the upregulation of CDKN2A mRNA expression in type II EC may increase tumour-adapted survival through the activation of autophagy, suggesting the heterogeneity of autophagy regulatory mechanisms across subtypes (150, 151).

In nutrient-deprived or hypoxic microenvironments, moderate autophagy maintains tumour cell survival through the removal of damaged organelles. However, excessive autophagy triggers programmed cell death (147). This bidirectional regulatory mechanism is particularly significant in the Beclin1 expression pattern: high Beclin1 levels are correlated with type I EC tissue grade, myometrial invasion, and poor prognosis (152), and the mechanism may involve HIF-1α-mediated protective autophagy (153). However, other studies have shown that Beclin1 and PTEN are downregulated during normal–carcinoma transformation and that their expression is correlated only with the degree of tissue differentiation (154).

Targeted therapeutic strategies based on autophagy modulation present important translational value. Inhibitors of the PI3K/AKT/mTOR pathway, such as diosgenin (155), induce pro-apoptotic autophagy through the activation of p53 signalling, while chemotherapeutic agents, such as cisplatin (155), trigger protective autophagy by inhibiting this pathway.

Studies on resistance mechanisms have shown that reactive oxygen species-dependent autophagy activation is significantly enhanced in paclitaxel-resistant cell lines (HEC-1A/JEC) and that the resistance phenotype is reversed by treatment with chloroquine or Beclin1-shRNA (156). MIR218 expression is significantly downregulated in Ishikawa and RL95–2 paclitaxel-resistant cells. Restoring MIR218 expression enhances the sensitivity of resistant endometrial cancer (EC) cells to paclitaxel through interactions with the 3’-UTR of HMGB1. This interaction directly targets HMGB1, which is upregulated in paclitaxel-resistant EC cells and contributes to chemotherapy resistance by mediating autophagy (157).

Novel metabolic modulators provide new ideas for EC treatment: metformin induces autophagy-dependent apoptosis via the AMPK/mTOR axis (158), whereas the GLP-1 receptor agonist liraglutide enhances autophagy to improve the endometrial microenvironment via the AMPK–TGFBR2–SMAD2/3 pathway. These findings establish a mechanistic basis for designing autophagy-targeted precision therapies (159, 160).

### 4.2 Ovarian cancer

OC is the most lethal malignant tumour of the female reproductive system, with epithelial carcinoma being the predominant histological type, accounting for approximately 80% of cases (161). Current research indicates that the function of autophagy in OC changes depending on the stage of the disease (**Figure 5**).

In benign or FIGO stage I/II ovarian cancer, autophagy inhibits tumorigenesis by removing aberrant proteins and damaged organelles. High expression of LC3 and Beclin1 is correlated with a good prognosis, suggesting that autophagic activity may delay malignant transformation by maintaining mitochondrial homeostasis and genomic stability (162, 163). In the microenvironment of progressive tumours or under chemotherapy, autophagy provides cancer cells with energy substrates such as amino acids and fatty acids through catabolism, facilitating their adaptation to hypoxia and nutrient deficiency stress (164). This adaptive mechanism is crucial for cancer cells to survive and proliferate under such harsh conditions.

Notably, reduced Beclin1 expression is significantly associated with platinum resistance and may lead to the accumulation of the antiapoptotic protein Bcl-xL through the disruption of autophagic flux (165). As a key bridging protein in the autophagic degradation pathway, the expression level of p62 reflects the status of autophagic activity. Clinical cohort studies have confirmed that high cytoplasmic p62 expression in patients with ovarian plasmacytoid carcinoma is significantly associated with shorter overall survival (166). Mechanistic studies have shown that aberrant accumulation of p62 in chemotherapy-resistant cell lines inhibits autophagic death via a p53-independent pathway and that p62 knockdown reverses this phenotype (167, 168).

miRNAs significantly influence disease progression by targeting and modulating autophagy-related genes, such as mTOR and the ATG family. Recent studies have shown that miRNAs can alter the radiosensitivity of OC cells (169). For instance, in OC cells exposed to radiation, the downregulation of miRNA-4478 over time is associated with poor patient prognosis. Conversely, upregulation of miRNA-4478 expression can increase radiosensitivity by decreasing protective autophagy through the inhibition of FUS activity (170).

Recent studies have demonstrated that miRNA-214 increases radioresistance in OC by downregulating PTEN expression and subsequently activating the PI3K/AKT pathway (171). Additionally, investigations into chemoresistance mechanisms have revealed that miR-30a can increase cisplatin sensitivity by inhibiting the TGF-β/Smad4 pathway (172).

In ovarian cancer, the PI3K/AKT/mTOR pathway is aberrantly activated in approximately 60% of cases. This dysregulation can inhibit the initiation of autophagy through phosphorylation-mediated inactivation of ULK1 (14, 173). Preclinical studies have shown that BKM120 (a PI3K inhibitor) and everolimus (an mTOR inhibitor) induce autophagy, inhibit tumour growth and partially reverse chemoresistance. Activation of p38 MAPK or JNK induces protective autophagy, whereas inhibition of JNK3 enhances the therapeutic response through proautophagy-dependent death (174), highlighting the temporal and spatial heterogeneity of pathway regulation (175, 176).

Autophagy in the tumour microenvironment involves the release of IL-6 from CAFs to promote cancer cell invasion and autophagy activation via STAT3 signalling (177), and the natural compound resveratrol enhances autophagy-associated apoptosis through inhibition of STAT3 transcription (178), providing a new strategy for combination therapy.

### 4.3 Cervical cancer

CC, a significant gynaecological malignancy, is primarily driven by persistent infection with HPV, which can lead to cervical intraepithelial neoplasia (CIN) and subsequent malignant transformation (179). Recent studies have shown that autophagy plays a context-dependent dual role in CC, influencing malignant transformation, treatment resistance, and prognosis (**Figure 5**).

In the early stages of tumours and precancerous lesions, autophagy plays a protective role by selectively eliminating damaged mitochondria and abnormal proteins. Clinical histopathological analyses have revealed that the expression of autophagy-related proteins, such as Beclin1 and LC3B, is significantly downregulated in squamous cervical cancer (SCC) tissues. This downregulation suggests that inhibition of autophagic activity may be involved in the process of malignant transformation (180). *In vitro* experiments have demonstrated that overexpression of Beclin1 in HeLa cells can inhibit cell proliferation by restoring autophagic flux. Additionally, delayed tumour growth has been observed in a nude mouse xenograft tumour model (179).

Under tumour progression or chemotherapeutic intervention conditions, however, autophagy maintains tumour cell survival through metabolic reprogramming mechanisms. Abnormal accumulation of p62 protein in drug-resistant cell models can mediate cisplatin resistance by blocking the autophagy-dependent apoptotic pathway (181). Notably, the cyclopiroxamine-induced PARK7/ROS signalling axis triggers glycophagy inhibition, glycogen aggregation, and glycogen aggregation-mediated inactivation of YAPI, providing a potential biomarker for monitoring its efficacy (182).

Epigenetic regulatory networks play important roles in autophagy-related signalling pathways. tET1 gene deletion promotes autophagic activity by activating the mTOR–RAP1 signalling axis, which enhances EMT and metastatic potential. Genome-wide methylation analysis revealed that aberrant epigenetic modifications of autophagy-related genes, such as ATG5 and LC3, may represent novel targets for prognostic assessment (183). Furthermore, the NUPR1–TFE3 axis was shown to promote autophagy-dependent CC cell proliferation through activation of the PI3K/AKT/mTOR pathway, and knockdown of NUPR1 expression significantly inhibited the growth of transplanted tumours and increased the apoptosis rate (184).

New advances in optimization studies have targeted therapeutic strategies for CC. Curcumin combined with shRNA-mediated ATG3 gene silencing synergistically inhibits the migratory ability of HPV-positive cells (SiHa/HeLa), and the mechanism involves the inhibition of MMP2 expression and the enhancement of ATG3-dependent autophagic flux regulation (185).

In the context of radiation therapy, fundamental research has revealed that autophagy activation mediated by the Akt/mTOR pathway promotes the formation of multinucleated giant cells (PGCCs), thereby contributing to radioresistance. Conversely, the application of autophagy inhibitors has been shown to effectively increase the sensitivity of cancer cells to radiotherapy (186). These insights provide a theoretical foundation for the development of combination therapies involving natural drugs and radiosensitization strategies that specifically target autophagy.

### 4.4 Uterine fibroids

Uterine fibroids (UFs) is a benign tumour that occurs in the uterine muscle layer, which is characterized by the abnormal proliferation of smooth muscle cells and fibroblasts, as well as the pathological deposition of fibrotic extracellular matrix (187). These tumours can cause serious health problems in women, including heavy menstruation, pelvic pain and reproductive dysfunction (188).

Abnormal oestrogen signalling is considered a central driver of UF progression. In addition to directly stimulating smooth muscle cell proliferation, oestrogen can remodel the tumour microenvironment by inducing the expression of fibroblast-activated protein (FAP), which not only promotes the abnormal deposition of extracellular matrix components (e.g., collagen) but also regulates the release of growth factors, such as TGFβ and IGF-1, accelerating the transdifferentiation of fibroblasts to myofibroblasts (189, 190).

Molecular mechanism studies revealed that FAP can deregulate the negative regulation of core autophagy molecules (e.g., mTOR) by downregulating Akt levels through the PI3K/Akt signalling axis, which provides experimental evidence for the regulation of autophagic homeostasis by the oestrogen–FAP pathway (191).

Autophagy is associated with significant abnormalities in UF development, and studies have confirmed that downregulation of ATG4D expression promotes rhabdomyosarcoma cell proliferation, which is correlated with increased fibrosis within rhabdomyosarcoma, suggesting that autophagy inhibition may be involved in tumour growth (192). Notably, the abnormal expression of ATG family proteins in gynaecological tumours is characterized by conserved regulation across tumour types, providing a theoretical basis for exploring autophagy-related therapeutic targets (192, 193).

In the context of therapeutic strategies for UFs, ulipristal acetate (UPA) has emerged as a promising selective progesterone receptor modulator. Clinical trials have demonstrated its efficacy in managing UF-related symptoms, with its therapeutic effects linked to the activation of the autophagy pathway (194). Specifically, UPA treatment has been shown to increase the LC3-II/LC3-I ratio, which is a hallmark of autophagy activation.

In addition, this will also lead to the accumulation of p62/SQSTM1 protein and the recovery of ATG4D expression (193). These molecular events together contribute to the inhibition of cell activity, which may explain the shrinkage of fibroid volume and symptom relief observed in patients. This mechanistic understanding lays the foundation for further for further exploring UPA as a therapeutic option for uterine fibroids and potentially other conditions where autophagy modulation could be beneficial (195).

These studies have established autophagy recovery and FAP inhibition as the two pillars of the next generation of UF therapy (**Figure 5**). Future research must focus on patient stratification, targeted delivery and mechanism-based combined treatment, so as to promote the clinical management of UF to achieve breakthroughs and go beyond the current limitation of relying only on hormone intervention.

## 5 Autophagy and tumour drug resistance

Autophagy significantly contributes to neoplastic cell adaptation to chemotherapeutic agents, molecularly targeted drugs, and radiation treatment, establishing itself as a major mechanism underlying treatment failure. This cytoprotective function operates through diverse molecular cascades, such as AMPK, HSF1, and reactive oxygen species signalling. Therefore, concurrent administration of autophagy-suppressing compounds with established anticancer regimens represents a strategically viable approach to overcome therapeutic resistance and improve clinical outcomes.

Extensive experimental support characterizes autophagy as a central biological process enabling therapeutic resistance in cancer cells. Studies have shown that overexpression of DNAJC5 regulates autophagy by upregulating the expression of proteins in the BiP-IRE1α-XBP1 endoplasmic reticulum stress pathway, thereby promoting resistance to cisplatin in ovarian cancer (196). Furthermore, knocking down miR-675-5p inhibits the mTOR signalling pathway and autophagy via TSC2, thereby reducing the resistance of OC cells to cisplatin; this may represent a potential therapeutic target for ovarian cancer (197).

In patients with endometrial cancer, IKBKE knockdown significantly reduces the proliferation and migration of progesterone-resistant EC cells. Treatment with CYT387 (a selective IKBKE inhibitor) inhibits autophagy activity and reduces cell viability, suggesting a potential molecular target for overcoming progesterone resistance (198).

Clinical evidence confirms that adding the autophagy inhibitor chloroquine can reverse this resistance (199), indicating that blocking autophagy sensitizes tumour cells to chemotherapeutic agents. Sustained tumour growth suppression is achieved only when kinase inhibitors and autophagy inhibitors are used concomitantly. Thus, the observed clinical efficacy in these studies stems from overcoming pre-existing drug resistance, rather than from direct sensitivity to the autophagy inhibitor itself. This conclusion requires further validation through pharmacodynamic evidence of autophagy suppression in tumour biopsy samples.

Inhibiting autophagy can counteract various molecular mechanisms driving resistance to BRAF mutations. Current clinical trial results related to autophagy and drug resistance demonstrate that autophagy inhibitors can effectively overcome tumour cell resistance to kinase inhibitors (200). A randomized phase II trial (NCT05063812) in platinum−sensitive relapsed OC demonstrated that adding hydroxychloroquine to chemotherapy enhances chemosensitivity. Hydroxychloroquine suppresses autophagy by blocking autophagosome–lysosome fusion (201).

Autophagy can induce tumour cell resistance to multiple chemotherapeutic agents. For instance, OC cells develop resistance to the cytotoxic drug paclitaxel through autophagy (202). In CC, the knockdown of ClC-3 reverses cisplatin resistance through Akt/mTOR-dependent autophagy (203), while Sesamol effectively targets EPHA2 to enhance cisplatin sensitivity. Knockdown of EPHA2 can enhance mitochondrial fusion and inhibit autophagy activity, thereby restoring the sensitivity of drug-resistant cells to cisplatin (204). In endometrial cancer, autophagy maintains the characteristics of cancer stem cells that drive multidrug resistance, and liposomal Resvida shows promise in regulating chemotherapy resistance by regulating the expression of autophagy-related genes (205).

Cumulative investigative data confirm that autophagy-directed approaches are strategically valuable for reversing oncological treatment refractoriness. Detailed characterization of autophagy-mediated resistance mechanisms will correspondingly generate foundational insights to guide subsequent therapeutic development.

AMPK, a central regulator of cellular energy and metabolism, is involved in various tumour cell physiological processes, including autophagy (206). Consequently, it serves as a critical entry point for studying the role of autophagy in tumour resistance mechanisms. Hunger-induced activation of AMPK triggers autophagy-dependent cell migration through the ULK1-SH3PXD2A-MMP14 pathway, highlighting the connection between metabolic stress and the invasive phenotype of ovarian cancer (207). TRP14 promotes cisplatin resistance by inducing autophagy through the AMPK/mTOR/p70S6K signalling axis, while costunolide (CTD) inhibits the autophagic flux by inactivating the AMPK/mTOR signalling pathway, thereby increasing the sensitivity of ovarian cancer cells to cisplatin (208, 209). Therefore, targeting the AMPK-autophagy axis is a potential strategy for intervening in the progression of ovarian cancer.

Furthermore, heat shock factor protein 1 (HSF1), a master transcriptional regulator of cellular stress responses (210), can directly bind to the promoter of the core autophagy gene ATG7. This binding upregulates ATG7 expression, thereby activating autophagy and promoting tumour cell drug resistance (211). However, as HSF1 regulates drug resistance through multiple mechanisms, the efficacy of autophagy-based therapeutic strategies that solely target the HSF1-ATG7 axis requires further validation.

Many anticancer treatments prompt tumour cells to generate ROS, which are significant inducers of autophagy. Numerous studies have elucidated the crucial mechanisms through which ROS-mediated autophagy promotes tumour drug resistance. In ovarian cancer, the overexpression of ferritin-like protein 1 (FDX1) drives paclitaxel resistance through copper-induced ULK1/ATG13-mediated autophagy, and this mechanism is closely related to the increase in ROS levels (212). In low-grade serous OC with KRAS mutations, DIRAS3 triggers ROS-mediated apoptosis by downregulating NFE2L2/Nrf2 transcription, reducing antioxidant levels, and inducing oxidative stress while also inducing cell protective autophagy, thereby conferring adaptive resistance. This resistance can be reversed by chloroquine or DC661 treatment (63).

ROS simultaneously induces apoptosis and autophagy promoting survival in tumours. The treatment outcome depends on the balance between these two processes, and this balance varies significantly depending on the type of tumour and individual differences. In the future, more precise models are needed for quantitative analysis.

In CC, CPX induces protective autophagy dependent on reactive oxygen species through the PARK7–PRKAA1 or ROS–mTOR axis, thereby weakening its own antitumor efficacy. This self-limiting resistance can be alleviated by the reactive oxygen species scavenger NAC, which redirects the response to glycosylated autophagy-mediated growth inhibition (182). In endometrial cancer, the natural flavonoid compound MA triggers ER stress pathways by upregulating intracellular ROS levels, thereby triggering apoptosis and protective autophagy, suggesting that simultaneous inhibition of autophagy may further increase its antitumour efficacy (213). Emerging evidence indicates that the IRE1α-XBP1-autophagy axis promotes pathological progression under hypoxic stress, and targeting this cascade may counteract autophagy-mediated damage (214).

Collectively, autophagy is a key adaptive mechanism by which malignant cells evade treatment-induced cytotoxicity. Therefore, to effectively reverse drug resistance in a clinical setting, targeted strategies must be implemented, such as combining autophagy inhibitors (e.g. chloroquine/hydroxychloroquine) with standard anticancer therapies.

## 6 Targeting autophagy in cancer treatment

Inhibition of autophagy induces a series of adverse biological effects in tumour cells, including impaired mitochondrial metabolism, redox imbalance, and reduced energy supply, thereby suppressing growth and even triggering cell death. Consequently, numerous preclinical studies have provided a rationale for exploring autophagy-targeting strategies in cancer therapy.

Chloroquine (CQ) and hydroxychloroquine (HCQ) represent the most frequently employed autophagy inhibitors in clinical practice. These agents disrupt autophagic progression by preventing the fusion between autophagosomes and lysosomes. Additional research demonstrates that beyond their autophagy-blocking properties, CQ/HCQ can increase malignant cell susceptibility to chemotherapeutic agents through autophagy-independent mechanisms (215). For example, in the treatment of ovarian cancer, the combination of CQ and cisplatin was shown in preclinical models to reduce the proliferation of drug-resistant epithelial ovarian cancer cells and increase apoptosis, inducing DNA damage (elevated γH2AX) and G2/M phase arrest; knockdown of p21WAF1/CIP1 partially reversed this effect (216). In cells with low BRCA2 expression, CQ significantly enhanced the efficacy of cisplatin, inducing increased LC3-II expression and enhanced apoptosis in tumour tissue *in vivo (*217).

The therapeutic outcomes of autophagy-targeted interventions have shown inconsistent efficacy across clinical trials. In the treatment of cervical cancer, HCQ has been shown to inhibit autophagy by blocking the degradation of autophagosomes in SiHa cells, thereby promoting apoptosis (218). However, clinical data on the use of HCQ alone in gynaecological malignancies remain limited. *In vitro* studies have demonstrated that combining HCQ with itraconazole can synergistically induce lysosomal dysfunction and exert antitumor effects against epithelial ovarian cancer cells. Nevertheless, in the dose escalation experiments, this drug combination did not demonstrate any clinical anti-tumour activity (219). Additionally, CQ can suppress proliferation and induce apoptosis in various endometrial cancer cell lines by inhibiting autophagy. In cisplatin-resistant Ishikawa cells, sensitivity to CQ is actually enhanced due to their higher baseline levels of autophagy (220).

The above preclinical evidence provides a theoretical basis for the chemosensitizing effect of chloroquine. However, these studies are still limited to experimental systems. It is currently unclear whether the concentration of hydroxychloroquine achieved within tumours at a tolerable dose can produce sufficient autophagy inhibition. This issue needs to be further explored through pharmacological research.

In the field of ovarian cancer, the same Phase II clinical trial mentioned above found that adding hydroxychloroquine to chemotherapy did not improve survival rates. The results further emphasised the necessity of patient selection based on biomarkers (201). This may be due to the dynamic nature of tumour autophagy, which a single biomarker assay is unable to fully capture. Furthermore, the failure to stratify patients according to baseline tumour autophagy levels has also limited the results. In contrast, in a Phase I/II clinical trial for advanced endometrial cancer, ABTL0812 (ibrilatazar) was safe and showed encouraging but preliminary efficacy when combined with paclitaxel and carboplatin. By inhibiting the Akt/mTOR pathway to induce cytotoxic autophagy, this drug was associated with disease control in a majority of evaluable patients; however, confirmatory randomized trials are needed to establish its clinical value (221).

Additionally, the maximum tolerated dose (MTD) of HCQ varies depending on its concomitant medications, complicating dosing regimens. In an I-phase trial involving 22 patients with advanced solid tumours (including gynaecological tumours such as ovarian cancer), when HCQ was used in combination with carboplatin and gemcitabine, its MTD was only 100 mg per day (222). In an I-phase clinical trial for patients with solid tumours, when hydroxychloroquine was used in combination with high-dose intensive temozolomide, the MTD (maximum tolerated dose) was 400–1200 milligrams per day (223). Thus, the tolerated dose of HCQ varies substantially with the combined medication, chemotherapy intensity, and tumour type. However, whether MTD achieves sufficient intratumoral autophagy inhibition remains unclear, as the drug concentration within tumour tissues and the autophagy markers are usually not directly measurable.

In addition to conventional treatment methods, more and more evidence indicates that certain targeted therapies can promote the degradation of PD-L1 through selective autophagy mediated by p62, thereby exerting immunomodulatory effects (224). In a xenograft model of clear cell ovarian cancer, it was found that using Lys05 to inhibit autophagy significantly enhanced the cell death induced by sunitinib. Moreover, in patients with recurrent endometrial cancer, sunitinib monotherapy showed promising but short-term efficacy in a Phase II trial, with manageable toxicity, which requires further research on combined treatment strategies (225). This provides a plausible mechanistic explanation for the immunostimulatory effects of sunitinib, suggesting that certain targeted therapies may exert immunomodulatory effects by modulating autophagy. However, direct evidence linking this autophagy-dependent mechanism to survival benefits is lacking.

In the field of CC, preclinical studies have shown that the combination of the PI3K inhibitor BKM120 with hydroxychloroquine enhances apoptosis and inhibits the proliferation of PIK3CA-mutant cell lines, providing a preliminary theoretical basis for the clinical evaluation of this dual-targeted therapeutic regimen in patients with PIK3CA-mutant CC (226).

In gynaecologic oncology, therapeutic vaccines based on autologous tumour cell lysates or specific cellular components (e.g., autophagosomes) represent a strategy to activate antitumor immunity. For instance, in preclinical models of ovarian cancer, a vaccine using autophagosome-enriched tumour lysates to sensitize dendritic cells can induce a stronger antigen-specific T-cell response (227). However, the preparation of such individualized vaccines takes a long time, which poses a practical obstacle to rapid treatment for advanced patients (228). Non-autophagic strategies, such as electroacupuncture, can also reshape the tumour immune microenvironment by preserving bone marrow function, providing additional combination therapy options (229). Taken together, this approach remains highly experimental.

In summary, the therapeutic strategies for autophagy in gynaecological cancers are still in the early stage of clinical research. Future research progress requires improving the design of clinical trials, establishing validated biomarkers for drug efficacy, and deeply understanding the scene-dependent nature of autophagy function. Only in this way can personalized autophagy regulation strategies be developed for specific patient subgroups and types of gynaecological cancers.

## 7 Conclusions and perspectives

This comprehensive review systematically examines the dual regulatory roles of autophagy in gynaecological tumour biology, with a particular focus on its context-dependent functions across tumour initiation, progression, and therapeutic resistance. Autophagy serves as a critical cellular homeostasis mechanism that can either suppress tumour development or promote established malignancies, with its net effect determined by tumour type, stage, genetic background, and microenvironmental factors.

This review addresses a significant knowledge gap by synthesizing the complex, often contradictory, functions of autophagy in female reproductive system cancers through a unified framework that integrates molecular mechanisms, immune modulation, and therapeutic applications. The identified patterns of autophagic regulation provide valuable insights for developing targeted cancer therapies. In addition, we highlight promising therapeutic targets for the treatment of gynaecologic malignancies across multiple malignancy types. Elucidation of the role of autophagy in modulating immune checkpoint molecules and tumour-infiltrating immune cells provides a mechanistic foundation for combining autophagy modulators with immunotherapy.

This review excels in synthesizing evidence on autophagy across mechanisms, experimental models and cancers types; however, its interpretation is constrained by reliance on preclinical systems that poorly reflect human heterogeneity, the absence of clinical autophagy–flux assays, and limited subtype-specific or tumour-selective insights. Trials with broad use of autophagy inhibitors have shown only modest benefits. Future priorities should therefore focus on patient-derived organoids, humanized mice, noninvasive flux monitoring (imaging or circulating biomarkers), and context-specific modulation tailored to cancer type, genetics and treatment stage.

Through systematic resolution of the documented research challenges, we can optimally integrate emerging autophagy-targeting strategies with conventional chemotherapy, tumour-suppressing medications, and immunotherapeutic approaches, thereby expanding their clinical implementation in gynaecological cancer management.
